# Strategy and Decision Making in Karate

**DOI:** 10.3389/fpsyg.2019.03025

**Published:** 2020-01-17

**Authors:** Jérôme Frigout, Sophie Tasseel-Ponche, Arnaud Delafontaine

**Affiliations:** ^1^I3SP Laboratory, Department of Sports Science and Physical Education, Paris Descartes University, Paris, France; ^2^Médecine Physique et de Réadaptation, CHU Amiens-Picardie, Amiens, France; ^3^EA 4559, Laboratoire de Neurosciences Fonctionnelles et Pathologies, Centre Universitaire de Recherche en Santé, CHU Sud, Amiens, France; ^4^CIAMS, Université Paris-Sud, Université Paris-Saclay, Orsay, France; ^5^CIAMS, Université d’Orléans, Orléans, France

**Keywords:** karate, modeling, inner logic, strategy, decision making

## Abstract

Karate will be included in the 2020 Summer Olympics in Tokyo as an additional sport. The inner logic of this activity includes a specific scoring system and way of modeling. Three hundred and nine bouts were observed in the competition context, which resulted in new perspectives on training and competition. The scoring of punches (43.7% of total scored points) and face kicks (37.9%) appears to be more significant (*p* ≤ 0.01) than that of body kicks (15.3%, *p* ≤ 0.01) and leg-sweeping (3.1%, *p* = 0.31). Penalties appear to be very significant and associated with victory when “scored” by the competitor against himself or herself (*p* ≤ 0.01). Competitors must score points and penalties. This zero-sum game induces a simple rivalry, whose purpose is domination and which must rely on a predefined strategy and initiative. Karatekas have to make decisions, such as when taking the risk to score points and penalties, whether or not they lead the score. Karatekas may decide to expose or protect themselves, create situations, or simply remain realistic and adhere to the plan. The question of decision making, which is central to this work, forces us to focus our future work on the notions of expectations and self-fulfilling prophecies.

## Introduction

Karate is a traditional martial art that originated in Japan. It is also a sport that will make its first appearance in the Olympic Games programme in Tokyo 2020.

According to the 2016 Referee rules (FFKDA, 2016) of the French Karate and Related Sports Federation [*Fédération Française de Karaté et Disciplines Associées* (FFKDA, 2016)], the bouts are managed by a central referee, who applies penalties, and by four judges seated at the corner, who record the points awarded. The different types of scores are as follows: *Yuko* for a 1-point awarding punch, *Waza ari* for a 2-points awarding upper body kick, and *Ippon* for a 3-points awarding face kick or a leg-sweeping technique followed by a punch or a kick. In the case of a tie at the end of the regular time, the referee and judges grant a flag in favor of the winner (fighting spirit and strength, superiority of tactics, and techniques displayed, initiative of actions). For a point to be awarded, four criteria must be met (potential effect, awareness, timing, distance), and at least two flags must be raised in favor of the competitor’s colors (blue or red); the four judges may simultaneously award points to each competitor (for instance, two flags for the competitor with the blue belt, i.e., *ao*, and two flags for the competitor with the red belt, i.e., *aka*). The central referee may impose penalties (category 1 for excessive contact, category 2 for prohibited behavior), which must be confirmed by the four judges. When points are awarded or penalties imposed, the bout will stop. Thus, the fight occurs in segments, and the competitors take their original positions before the bout resumes. If a competitor has a clear lead of eight points, the bout is stopped, and victory is awarded.

Three additional rules must be followed: The “ten-second” rule allows the referee to withdraw a competitor from the tournament who is knocked down, who fell, or who is thrown, and who does not get back up on his or her feet within a maximum of 10 s. In addition, the use of video review allows the coach, seated in the corner of the competitor, to request a review of a decision and be awarded points despite the observation and confirmation of the judges. Finally, the *senshu* rule (the competitor who has obtained the first unopposed score advantage at time-up), which is currently being tested in international competitions, has just been introduced for the very first time at the 2017 Open Paris Karate–Premier League, held between 27 and 29 January at the Pierre de Coubertin stadium.

The goal of our study is to determine whether, beyond potentially modeling karate competitions, it would be possible to issue coaching requirements. We assume that the existing literature on the scoring system in karate must be supplemented by issue related to the expectations of the coaches (Fantoni, 2016) and the relationships between the coaches and their competitors (K/Bidy and Escalie, 2016). What types of motor decisions could karatekas make during a competition in an ongoing bout and in their relationship with their coaches (Biéchy, 2012; Fantoni, 2016)?

To achieve this goal, the authors carried out observations during four selective competitions at the French district level (in the cities of Rungis, Thiais, and Orly, all located near Paris, in the Val-de-Marne Department), which may lead competitors to participate in national championships in their own categories in case they are successful (FFKDA, 2016). Additionally, two competitions at international level were observed: the Open Paris Karate–Premier League, which is a ranking competition that allows competitors to score points to be qualified for the 2020 Olympics in Tokyo.

For instance, Table 1 shows quantitative information on a bout that occurred at the 2017 Open Paris Karate–Premier League, on January 29, during one final bout.

**TABLE 1:** Quantitative information on one final bout, 2017 Open Paris Karate–Premier League.

**Anonymous final – 2017 Open Paris Karate-Premier League**					
***Aka* (red color): victory 4**			***Ao* (blue color): defeat 1**		
**Simultaneous actions**	**Attempted**	**Scored**	**Simultaneous actions**	**Attempted**	**Scored**
*Yuko*	12	1/0	*Yuko*	14	1/0
*Waza ari*	3	0	*Waza ari*	1	0
*Ippon*	6	1/0	*Ippon*	5	0
Leg-sweeping *ippon*	0	0	Leg-sweeping *ippon*	4	0
*Senshu*	N/A	1	*Senshu*	N/A	0
Category 1	N/A	0	Category 1	N/A	0
Category 2	N/A	3	Category 2	N/A	0
*Video review*	1	0	*Video review*	1	1

This bout analysis is clearly in favor of the results presented by Vidranski et al. (2015) concerning the significant differences between actions attempted and points scored in competitive karate.

Thanks to this type of observation, we were able to define a performance model in karate competitions (Frigout et al., 2017, p. 75) by using Biéchy’s (2012, p. 38) work: the technical and tactical paradox, which requires finding the right balance between “*exposing, realism, protecting, creativity*.”

To this end, the coach must act as a researcher and try to be as objective as possible through observation.

Modeling in sports is the subject of several studies that have proven relevant: from Franks and Goodman (1986), who define the necessity of a systematic model and quantification, to Garganta (1998), who discusses game modeling in the context of better training, to Hughes et al. (2012) in the context of soccer. These different works discuss how modeling “can be considered as a mediator between a theoretical and an empirical field (…) in order to analyse performance trends and to prioritize potential issue within the training structure” (Hughes et al., 2012, p. 403).

How should a coach choose a strategic training method specific to competition while implementing several tactical adaptation schemes? Should competitors try to simultaneously validate points to minimize both their gains and losses? Could the Nash (1950) be applied and used to model training sessions?

Let us remind ourselves that the *inner logic* (Parlebas, 2005) of sports relies, in regard to exclusive fighting sports, on the following relation: R ∩ S = Ø (Parlebas, 1986, p. 208). This relation reminds us that two opposing karatekas may not be simultaneously rivals and partners. This type of game where the scoring system is strictly contrary (Parlebas, 2005, p. 30) can be described by the following formula M^+^ = Ø. In a competition, karate is a zero-sum game (Rapoport, 1967). In addition, Nash (1950) provides evidence that there is no equilibrium in regard to pure strategy and that there is a need to rely on probabilities, i.e., a mixed strategy.

Thus, in relation to the new *senshu* rule mentioned in part 1, coaches and karatekas should not confuse an offensive with an initiative. Here, initiative in relation to formulating a strategy does not necessarily mean choosing to attack. Therefore, we must examine the notions of *decision making* and *rationality* (Shubik, 1971; Raïffa, 1973) and *expected utility* (Von Neumann and Morgenstern, 1944). These notions are linked to the concepts and possible scopes of game theory (Von Neumann and Morgenstern, 1944; Davis, 1973).

## Materials and Methods

Between January 24, 2016, and January 29, 2017, observations were carried out in real time by one of the authors, who is holder of a national degree (*BEES 2^è*me*^ degré karaté*), by using and filling out the official FFKDA game sheets during the bouts.

The data collection method used by one of the authors is not subject to any objective errors, as the conclusions are supported thanks to the electronic device present in the competitions (computer, scoring and video screens, timer), which indicate, during and at the end of each fight, every element of the scoring: points scored, penalties, *senshu*, and in specific cases the victory by flag vote (when no points are scored).

Additionally, as karate fights are stopped to deal with points and penalties attributions, the scoring registered in the specific sheets was possible to make with no mistakes. Every time a point or a penalty had to be scored, the fight was stopped: at that precise time, the author used the scoring sheets.

This organization of karate competitions always uses these kinds of electronic devices. These facilitate the scoring for every observer (the coaches, of course, and also the authors).

The observations were made during six different competitions with various types of opponents, such as children, teenagers, adults and veteran athletes (more than 55 years old) of both genders. A significant proportion of international athletes, including the top eight ranking karatekas in the last world championships, in each weight division and of both genders (Linz, 2016), were observed for a total of 191 fights. In addition, 118 bouts in the children, teenage and veteran categories for both genders were examined. A total of 148 bouts in the male category and 161 bouts in the female category were attended. A total of 618 observations in 309 bouts were made of competitors involved in a fight.

Four competitions were followed in the national categories (October 9, 2016, in Rungis, during which 66 observations were made of children; January 15–22, 2017, in Thiais, during which 80 observations were made of teenagers and seniors and 66 of children; January 29, 2017, in Orly, during which 24 observations were made of veteran athletes) and 2 competitions in the international categories (January 24, 2016, and January 28, 2017, at the Open Paris Karate–Premier League, during which six observations and 376 observations were made of seniors athletes). The study complied with the standards established by the Declaration of Helsinki. The participants were fully informed about the protocol before participating in this study and signed an informed consent form.

The experiment was approved by the local ethics committee of the University Paris-Saclay (affiliations: EA 4532; CIAMS, Université Paris-Sud., Université Paris-Saclay, 91405 Orsay, France; CIAMS, Université d’Orléans, 45067 Orléans, France).

These observations represented a total of 38.187% of national categories, including children, veteran athletes, teenagers and adults (seniors), and a total of 61.812% of adult (senior) international categories (Table 2).

**TABLE 2:** Informations concerning the categories of competitors observed.

**Categories**	**Male children**	**Female children**	**Male teenager**	**Female teenager**	**Male senior**	**Female senior**	**Male veteran**	**Female veteran**	**Total**
Age	6–11	6–11	12–17	12–17	18–34	18–34	35+	35+	NA
National competitions	88	44	32	38	10	0	24	0	236
Weight categories (in kg)	Po −30 Po −25 Pu −35 Be −30 Be −40	Po −25 Po −30 Po −35 Pu −30	Mi −45 Mi −50 Ju −55 Ju −76 Ju +76 Ju Open	Mi −45 Mi −50 Ju −59 Ju +59	Se −84	/	Ve −75 Ve −84 Ve +84	/	NA
International competitions	0	0	0	0	142	240	0	0	382
Weight categories (in kgs)	/	/	/	/	Se −75 Se −84 Se +84	Se −50 Se −55 Se −61 Se −68 Se +68	/	/	NA
Poussin (6–7) = Po/Pupille (8–9) = Pu/Benjamin (10–11) = Be/Minime (12–13) = Mi/Cadet (14–15) = Ca/Junior (16–17) = Ju/Senior (18–34) = Se/Veteran (35+) = Ve									

Observations were made of the different components of the scoring system and, in particular, the data that the judges and referees validate as part of this system: the date and location of the event, the observer, the category (age, gender, weight), the belt (blue or red), the points scored (simultaneously or separately), the category 1 or category 2 penalties, the possible flags (in case of a tie at time-up), the total number of points scored, the victory or defeat (including before time-up in case of a lead of at least 8 points between competitors), the potential disqualifications, the potential injuries, and the *senshu* (first unopposed point advantage).

The observations carried out during bouts between the abovementioned categories enabled us to determine whether the scoring system may significantly model karate techniques in competitions and the quest for performance and whether coaches and athletes should include additional data in their practice sessions on the basis of a performance model and regular observations: those related to the inner logic of the activity and their consequences for the purposes of scoring or achieving a flag. The significance threshold was set at *p* < 0.05. A chi2 test was performed over a series of the karatekas involved in competitions with Sphinx IQ2^®^, a quantitative data processing software.

## Results

The results described in the tables below specify the number of competitors involved. The losing competitor appears before the/symbol and the winner after. Table 3 presents an overview of the points and penalties scored in relation to victory.

**TABLE 3:** Points and penalties scored in relation to victory.

**Points**	**0**	**1**	**2**	**3**	**4**	**5**	**6**	**7**	**8**	**9**	**10**	**11**	***p***
*Yuko*	193/41	71/74	34/60	7/51	1/36	1/23	2/11	0/5	0/3	0/1	0/2	0/2	*p* ≤ 0.01
	VS	VS	VS	VS	VS	VS	VS	VS	VS	VS	VS	VS	

**Khi2 = 211.87; ddl = 11**													
**S, significant; FS, few significant; NS, non-significant; VS, very significant**													
**xxx/xxx = occurrences scored by *ao*/*aka* (the two opponents)**													
*Waza ari*	285/256 VS	10/40 VS	4/7 VS	0/5 VS	0/1 VS								*p* ≤ 0.01
	VS	VS	VS	VS	VS								

**Khi2 = 27.58; ddl = 4**													
*Ippon*	293/214 VS	15/69 VS	0/22 VS	1/4 VS									*p* ≤ 0.01
	VS	VS	VS	VS									

**Khi2 = 70.82; ddl = 3**													
*Leg-sweeping ippon*	306/303 NS	3/6 NS											*p* = 0.31
	NS	NS											

**Khi2 = 1.01; ddl = 1**													
**Penalties**		**0**		**1**		**2**		**3**		**4**		***p***	

Category 1		264/253		45/56		12/17		6/6		3/0		*p* = 0.17	
		NS		NS^∗^		NS		NS		FS		^∗^All categories	
				14/23								^∗∗^Seniors only	
				VS^∗∗^									

**Khi2 = 6.44; ddl = 4**													
**Not significant relation with Khi2, but correlation between the number of penalties and victory**													
Category 2		154/140		155/169		87/107		39/69		6/0		*p* ≤ 0.01	
		NS		NS		FS		VS		S			

**Khi2 = 20.87; ddl = 4**													
**Among the six competitors disbarred, two were for endangerment (*mubobi*) which resulted in injuries**													

This table first shows the maximum number of occurrences by type of points scored and presents a significant link with the victory or loss. It appears that the number of points scored, expressed as a number of occurrences, is highly correlated with the use of punches.

The difference of results by category (all categories and seniors only) reveals that the victory is achieved by obtaining the seventh point, no matter the technique used (the total number of points scored). This trigger, which implies an irrevocable win, can be expressed by significance *p* ≤ 0.01 TS (Khi2 = 311.75; ddl = 15) for all categories and by *p* = 0.00 TS (Khi2 = 205.26; ddl = 12) for the seniors only.

The results that relate to bouts won before time-up (8-point gap between the two competitors) show a significant difference between all the bouts observed and those with only seniors involved. The percentage of bouts won before time-up, which is low overall (10.7%), decreases even more (4.8%) in regard to seniors.

As for the type of points scored, and given that each technique grants a different number of points, we observe that punches account for 43.7% of points scored, body kicks for 15.3%, face kicks for 37.9% and leg-sweeping techniques followed by a kick or a punch when the opponent has fallen for 3.1%. Such information shows that punches and face kicks validate 4/5th of the points scored and that leg-sweeping techniques only represent a low percentage of total points scored.

This table also shows how category 2 penalties (prohibited behavior: grabbing without engaging in combat, escaping or stalling, exiting the competition area) are frequently used by competitors who have a significant advantage in the score to keep the opponent from engaging in viable combat or scoring back. These penalties are used by competitors until receiving three penalties (the fourth one means that they will be disqualified). A particular case, entitled *Mubobi*, is a warning about self-endangerment (for instance, a competitor who throws themselves at the opponent, by exposing his or her face to a counterattack without being able to block or avoid it). For seniors only, category 1 (a single penalty), i.e., excessive contacts and forbidden techniques, also shows a significant link to victory. These results (Table 4) also highlight a small number of injuries (two in seniors) as well as a small proportion of disqualified competitors (those who received four penalties), both in categories 1 and 2.

**TABLE 4:** Points simultaneously scored and the *senshu* rule in relation to victory.

**Points simultaneously scored**			**0**		**1**	**2**	***p***
*Yuko*			295/292		11/13 NS	3/4 NS	*p* = 0.85
			NS				
*Waza ari*			307/309		2/0 NS		*p* = 0.16
			NS				
*Ippon*			307/309		2/0 NS		*p* = 0.16
			NS				
*Leg-sweeping ippon*							Ø

***Yuko* Khi2 = 0.32; ddl = 2/*Waza ari* Khi2 = 2.01;**							
**ddl = 1/*Ippon* Khi2 = 2.01; ddl = 1**							
**Flag rule**	**0**	**1**	**2**	**3**	**4**	**5**	***p***

120 bouts analyzed	302/292 VS	3/0 VS	4/0 VS	0/4 VS	0/3 VS	0/10 VS	*p* ≤ 0.01
***Senshu* rule**							
189 bouts analyzed	0/9						*p* ≤ 0.01
	VS						

Flag rule Khi2 = 24.17; ddl = 5							
Columns indicates the flag vote number (0 means inequality of scores at the							
end of bouts. 1 to 5 means draws at the end of bouts, and so flag votes)							
*Senshu* rule Khi2 = 9.22; ddl = 2							
Only nine bouts finished with draws and so victories due to *senshu* advantage							

The results that we observed for potential points simultaneously scored by the two competitors are not significant: *p* = 0.85 NS for punches (Khi2 = 0.32; ddl = 2), *p* = 0.16 NS for body kicks (Khi2 = 2.01; ddl = 1), *p* = 0.16 NS for face kicks (Khi2 = 2.01; ddl = 1), and no occurrences for leg-sweeping techniques followed by a kick or a punch when the opponent has fallen. This is due to the number of points simultaneously scored, which is too low compared to all the points scored over all the competitions and bouts observed.

As for the *senshu* rule, we observed the following: before the rule was applied, only one competitor won his bout with the flags with a 0–0 score and another one only with the flags for an x–x score. A total of 120 bouts were analyzed in this case. With the *senshu* rule, no victory was awarded with the flags for an x-x score (a 0–0 draw or a draw with *senshu* scored simultaneously) and nine wins awarded with *senshu* for an x-x score. A total of 189 bouts were analyzed in the context of the application of this new rule. Additionally, no competitor lost this *senshu* advantage by being penalized in the last 15 s of the bout (this situation forfeits the *senshu* of the competitor who scores it). When *senshu* is awarded, at the end of a fight in an x–x situation (draw), the winner is the one who scored first, that is to say, he or she who obtained the *senshu* advantage. Each of these rules appears to be very significant in the way that it is applied: *p* ≤ 0.01 TS (Khi2 = 24.17; ddl = 5) for the flag rule in the case of a tie, and *p* = 0.01 TS (Khi2 = 9.22; ddl = 2) for the *senshu* rule and the application of the first unopposed point advantage in the case of a tie.

## Discussion

Several profiles of karatekas, whether male or female, are likely to win and have repeatedly done so. The karatekas can engage in offensive, defensive or countering tactics.

### Points Scored

There is a strong correlation between the number of *yuko* and victory. The more *yuko* a competitor wins, the larger the victory will be. This tendency reveals a possible paradox between fighting techniques that are based on single or cyclical (over two punches or kicks) actions and the tendencies observed, which show the significance of punches. What if the karateka had to combine two or three punches or kicks, just as boxers do, and only cease his or her sequenced cyclical actions and take his or her original position when the referee stops the bout?

In this scoring system, we notice that from 7 *yuko*, victory becomes certain in our entire sample. This certainty of scoring 7 *yuko* or more happened in this study to only 13 competitors (see Table 3). A *yuko* is a trigger in itself, both in terms of losses and gains.

Being awarded *waza ari* shows an even stronger correlation with winning than *yuko*. Surprisingly, the victory percentage is higher for 1 *waza ari* than for 2. From 3 *waza ari* scored, victory becomes certain. However, very few of these *waza ari* are scored. This could be explained by the body protection system the competitors use by placing their guards with their arms close to their bodies, which protects from body kicks and enables the opponent to counter the attack or to strike back. This could also be explained by the strong expectations of the referees and judges as to the scoring criteria (potential effect, awareness, timing, distance) or by a combination of these factors.

While only one competitor who was awarded 1 *ippon* lost in the entire sample of this study, managing to score with face kicking techniques significantly tends to lead to victory. This is something that coaches know in terms of points scored (3), and this imposes complex and hard work during physical training for athletes on one-foot postures when not protected against counterattacks or leg-sweeping techniques. This is also true for an *ippon* awarded for a leg-sweeping technique, which may lead to a loss in 33% of cases. This fact cannot be due to the risk of the technique itself, for if the *ippons* were scored, there would be no possibility of counterattacks because the bouts are stopped by the referee to score points. Rather, this must be due to other more complex relationships (i.e., were the sweeps tried as a desperate technical resource when a losing athlete tried to win back a losing match by earning the higher number of points with a single technique?).

In relation to the number of points scored, the results hold, yet again, few surprises: the more points that are scored, the most likely a victory is. A switch can be noted at 2 points. Defeat is then just as likely to occur as victory. A win becomes a certainty from 7 points, but there was 1 occurrence of 67 loses with at least 8 points (10–15). However, this result is significant.

### Penalties

Observing category 1 penalties (for excessive contact) independently does not show any significant link with victory, except for the fact that the fourth penalty results in the competitor being disqualified. Further, the likelihood of victory for a disqualified competitor is zero.

Observing these penalties as a whole, as Table 3 summarizes, is more interesting. Even if the link is not significant for khi2, there is an actual relation between the data. Victories are more or less frequent depending on the number of excessive contact penalties. The more contact penalties that are scored, the more victories that are obtained. Of course, the number decreases at 4 because of the resulting disqualification. However, the number of affected individuals is not high, as these penalties are moderately rare. As these components are not significant, looking for excessive contacts is not necessary, and we would rather elaborate on significant criteria that tend to lead to victory. By only examining the seniors and category 1 penalties (excessive contacts), it appears that contact penalties do not help competitors win, except for those who only receive penalty.

As for category 2 penalties (prohibited behavior), they become intricately linked to victory from the third penalty imposed. The penalties are not prior to the third strike and, because of the disqualification rule, automatically result in defeat when reaching the fourth penalty.

In all the results observed, only two competitors were disqualified because of prohibited behavior (exposing without protecting, called *mubobi*), which resulted in injuries. These two injuries affected karatekas who threw themselves into an offensive without protection to catch-up and who were hit by their opponents. These specific cases are among the few disabling actions authorized in karate competition (knock-out). In such circumstances, scoring a point requires meeting the four criteria (potential effect, awareness, timing, distance), as described in part 1, for the competitor who hit the wounded competitor. This is a situation that may happen to a led competitor. Thus, no tactics can effectively provoke the *mubobi* situation, which is almost always an accident, in which the motor control of one of the karateka is overwhelmed by an irrational action of the opponent.

### Points Scored Simultaneously, Flags and *Senshu*

While karate is a zero-sum game (Rapoport, 1967), as Parlebas (2005) specifies, karate may also be exercised, in certain circumstances, according to a cautious strategy, based on observing and waiting for the opponent’s moves. Attempting to minimize one’s gains for the purposes of minimizing one’s losses becomes a mostly defensive strategy. Can this be done?

If we agreed that such an exercise has already enabled an athlete to rise to the top of the world (for example : Nadége Aït-Ibrahim, karate World champion in 2012) or continental (for example : Anne-Laure Florentin, karate European champion in 2017) championships, i.e., that a Nash (1950) can be reached between two offensive strategies in a zero-sum game, the result would be considered suboptimal (Parlebas, 2005, p. 35) according to game theory.

Indeed, two of the rules of karate competitions result in cooperative strategies, thus becoming not significant: first, the stopping of the bout to award points to the first unopposed point advantage becomes a material standard, contrary to scoring simultaneous points, which, as we observed, is too rare of a situation to lead to any modeling that coaches and athletes could use. Scoring points during simultaneous actions is not enough to ensure a victory. Observations tend to lead to a contrary statement, although there are too few cases for that to be significant. Finally, the introduction of the *senshu* rule did not materially change the competitors’ behavior by making them unflinching attackers. However, recent studies on the number of valid points scored in karate competition point to the fact that having to balance the risk of obtaining a double advantage with the first unopposed score versus the fact that the opponent could be the one who obtains the valid score if the attack is not precise enough and is counterattacked has led to a delay in the moment of the first combat attacking actions.

In addition, the low number of points scored during the bout should not hide the reality of interactions between the opponents: many decisions are made and many actions are attempted by competitors concerning “*realism, creativity, exposing, protecting*” (Biéchy, 2012) during fighting situations.

Points were scored by attacking, defending, or counterattacking, and very few of the points were awarded simultaneously.

Given the quantitative information described in Table 1 of this article as to the bout between *Aka* (red color) competitor and *Ao* (blue color) competitor, we can highlight that *Aka* acts depending on what the scoring system analyzed considered significant (types of points, use of penalties, win at time-up) and is able to determine the errors that *Ao* makes (overuse of leg-sweeping techniques, failure to rely on penalties). *Aka* is well known for his *ippon* technics. Did *Ao* want to counter *Aka*’s work by using *ippon leg-sweeping* technics to prevent him from scoring with face kicks (even if he failed trying to do so)? Such quantitative information allows us to assume that there is a need for competitors to multiply social and motor actions. At that level of competition (finals of an international tournament), *Ao*’s fighting approach does not seem *realistic* enough and leads him to *expose* himself in vain. In contrast, *Aka*’s strategy, which is more appropriate considering the significance of the scoring system used, is closely related to the types of karatekas who often win. Additionally, in any combat, the technical level of both contestants might not be the same, and so one of them might have a clear advantage over the other, which cannot be reversed by any tactic.

The bout that we observed is a good representation of a zero-sum game, in which seeking an equilibrium between gains and losses is suboptimal.

These results may also be cross-checked with previous results from soccer (Hughes et al., 2012). Five purposes were discussed: “*movement analysis, educational use for coaches and players, tactical evaluation, database and technical evaluation.”* In comparison with the present work, the player roles were studied with educational and performance purposes, and we present the competitor roles concerning “*realism, creativity, exposing, protecting.*” Additionally, the significant results we present in this article concerning the scoring system in competitive karate and consequently the strategies that we propose next may be the first approach to identifying the Key Performance Indicators (KPI) used by Hughes et al. (2012). Indeed, further work may be engaged in to create a useful KPI for karate competitors.

Taekwondo researchers and trainers have already started working on modeling to “*provide objective data on competitive behavior*” (Menescardi et al., 2019). Their study focuses on technical and tactical behaviors and the regulations that need to be made concerning their inclusion in trainings, and of course, in bouts. The researchers assess that psychologists and coaches may help trainees pattern visualization plan sessions during training and thus elaborate on the tactical strategies to be used during competitions.

These previous studies may be of help for the continuation of this work.

On the basis of interactive logic, and as Conte and Lukonaitiene (2018) discussed regarding basketball and the case of different strategies, as to whether a team is winning or losing a match, we make some proposals that are the result of Biéchy’s interpretations, in which penalties may be scored by a competitor because he or she has not yet scored 3 penalties (Table 5).

**TABLE 5:** Possible strategic options when penalties may still be scored by a competitor.

**Current situation**	**Penalties**	**Possible strategic actions**
Leading competitor: suppressing valid touch surfaces	Category 1: less than three penalties imposed	– Defending + face punch (*jodan uchi*)
	Category 2: less than three penalties imposed	– Grabbing (+defending with face punch – *jodan uchi*) – Exiting the area + counter face kick (*jodan geri*)
	*Atoshi baraku* (last 15 s): less than three penalties imposed	– Defending + grabbing + face punch (*jodan uchi*) – Exiting the area + counter body or face kick (*chudan geri* or *jodan geri*)
Led competitor: entering – exiting – re-entering the touch distance	Category 1: less than three penalties imposed	– Attacking with a face or body punch (*jodan uchi* or *chudan uchi*) + attacking (free technics) – Attacking with a face or body punch (*jodan uchi* or *chudan uchi*) + defending
	Category 2: less than three penalties imposed	– Attacking (free technics) + defending + grabbing
	*Atoshi baraku*: less than three penalties imposed	– Same tactic as above
Vocabulary used by referees (FFKDA, 2016): *jodan uchi* (face punch), *chudan uchi* (body punch), *jodan geri* (face kick), *chudan geri* (body kick), leg-sweeping technique Strategic possible actions: attacking (attempt to hit an opponent by taking the initiative), counter (create an attack in an opponent attack to regain initiative and score points by hitting first), defending (block, dodge, parry an attack), existing the area (getting out the fighting area to win time, and cause a change of strategy of the opponent that he/she does not rationally decide), and grabbing (grab an opponent in order to prevent him/her to produce an attack/counter-attack that may score points).		

We also propose some different tactics when penalties may not be scored again by a competitor because this competitor has already scored 3 penalties (Table 6).

**TABLE 6:** Possible strategic options when penalties may not be scored because three are already scored.

Leading competitor: suppressing valid touch surfaces	Category 1: three penalties imposed	– Defending with body punch (*chudan uchi*) – Defending with face kick (*jodan geri*)
	Category 2: three penalties imposed	– Counter-attacking with face kick (*jodan geri*)
	*Atoshi baraku*: three penalties imposed	– Counter-attacking with body kick (*chudan geri*)
Led competitor: entering – exiting – re-entering the touch distance	Category 1: three penalties imposed	– Attacking (free technics) + defending on two sequenced height levels (mixing face and body technics)
	Category 2: three penalties imposed	– Same tactic as above
	*Atoshi baraku*: three penalties imposed	– Same tactic as above
Vocabulary used by referees (FFKDA, 2016): *jodan uchi* (face punch), *chudan uchi* (body punch), *jodan geri* (face kick), *chudan geri* (body kick), leg-sweeping technique Strategic possible actions: attacking (attempt to hit an opponent by taking the initiative), counter (create an attack in an opponent attack to regain initiative and score points by hitting first), and defending (block, dodge, parry an attack).		

Before the end of the bout, when 15 s remain, the referee announces *Atoshi baraku*. This period immediately before the end of the fight is very important in terms of tactics, because *senshu* advantage can be lost during this period. The result of the fight could be reversed, and the competitors cannot avoid combat, which – to a certain extent – might give some small advantage to the competitor who is losing the fight, as his or her opponent is forced to confront the fight. Avoiding combat (retreats without effective counter, holds, clinches, or exits from the area) during *Atoshi baraku* is penalized with a category 2 penalty (3rd degree, or 4th if the competitor already scored 3 penalties, which means disqualification) and with the loss of this *senshu* advantage.

It appears, indeed more than ever, that the definition of strategies and the determination of possible tactical schemes occurs in the relationship between the trainer and the trainee and that the trainers rely on *rationality* (Shubik, 1971) in deciding how to train the trainee and compete.

For us, the main question to be asked is that regarding decision making, which was raised by Raïffa (1973). Coaches should help the trainees reach the relevant control and performance levels (Le Scanff and Legrand, 2004), i.e., to work toward improving their self-esteem. Self-esteem leads to the ability to set goals for oneself. Optimizing technical and tactical work for the purposes of reaching a certain level of performance will be, in close collaboration with the coach, one of the tools (Le Scanff, 2003) for managing emotions and stress in the context of practice and in the bouts themselves. In this context, the trainer will have to work toward helping the trainee acquire new skills in relation to expectations, prophecies and a possible Pygmalion effect, as Fantoni (2016) showed. Determining the potential expectations of the coach will be a factor in the decision making for the athletes and will allow them to avoid the “*motor suicide*” obstacle (Fantoni, 2016, p. 155).

There are indeed “*interactive dynamics*” (K/Bidy and Escalie, 2016, p. 61) to be implemented by the trainer and the trainee once modeling is included in the practice process, in order to “*schedule ordinary training sessions*” and create a performance project.

According to Menescardi et al. (2019), “*objective data regarding successful behavioral patterns, (…) are important for coaches and psychologists to train and develop psychological strategies to prepare athletes. For instance, they can be used to individualize training sessions, including visualization of specific combat situations. (…) Coaches may use these findings for specific tasks related to technical-tactical improvement of scoring effectiveness.*”

The results of our analysis enable us to understand that an equilibrium between gains and losses is not relevant in a karate competition. As a zero-sum game, domination prevails.

The scoring system described in our analysis reveals that there is a need for karatekas to understand the wide variety of actions that can be used to score points. If competitors have to score points with punches as well as with face kicks, karatekas will have to make decisions depending on their opponent’s level and will have to wonder if *realism* is to be favored over *creativity* or if they should rather *expose* or *protect* themselves. Far from being ignored and poorly considered, both categories of penalties should be used. Competitors must be acquainted with a possible scoring knockout if their opponents expose themselves. In all these different fighting scenarios, the sport is a lasting game, where the trainee has to expect fierce struggles that are rarely won before time-up.

## Conclusion

The presentation of the technical and tactical paradox (which requires finding the right balance between “*exposing, realism, protecting, creativity*”) in the context of the analysis of 309 karate bouts clearly indicates suggestions for coaches and athletes, for both training sessions and competitions, to avoid unsuccessful situations and to perform by winning fights, thanks to successful behavioral choices. The paradox concept is used to expose how the apparently sound principles of tactical options such as “*exposing, realism, protecting, creativity*,” which reason from acceptable premises, may be opposed to some of the techniques used to score and how this could lead to a conclusion that seems logically unacceptable or self-contradictory. These strategic choices are presented in this study. However, to pursue this research in future work, we could try establishing key performance indicators (KPI) to make the coach-trainee relationship even more efficient on the basis of significant technical and tactical decisions, leading to rationality. These future analyses may be realized for different competition issues and in different contexts, from national to international contexts and for different categories (age, sex, level).

## Data Availability Statement

The datasets generated for this study are available on request to the corresponding author.

## Ethics Statement

The studies involving human participants were reviewed and approved by the University of Paris-Saclay (EA 4532, CIAMS, Université Paris-Sud, Université Paris-Saclay, Orsay, France; CIAMS, Université d’Orléans, Orléans, France). The patients/participants provided their written informed consent to participate in this study.

## Author Contributions

JF designed the study and collected, analyzed, and interpreted the data. JF, ST-P, and AD drafted and revised the manuscript and tables and gave the final approval.

## Conflict of Interest

The authors declare that the research was conducted in the absence of any commercial or financial relationships that could be construed as a potential conflict of interest.
